# Real-Time Evaluation of Ground Insulation Degradation and Fault Warning Method Under Multiple Operating Conditions for Train Traction Drive System

**DOI:** 10.3390/s25051296

**Published:** 2025-02-20

**Authors:** Zhenglin Cheng, Kan Liu, Xueming Li, Shaolong Xu, Zhiwen Chen, Fengbing Jiang

**Affiliations:** 1 College of Mechanical and Vehicle Engineering, Hunan University, Changsha 410082, China; chengzl@hnu.edu.cn; 2 CRRC Zhuzhou Electric Locomotive Institute Co., Ltd., Zhuzhou 412001, China; lixm10@csrzic.com (X.L.); xusl@csrzic.com (S.X.); jiangfb@csrzic.com (F.J.); 3 School of Automation, Central South University, Changsha 410083, China; zhiwen.chen@csu.edu.cn

**Keywords:** decision tree classification, degradation degree evaluation, fault mechanism, ground fault, multiple operating conditions

## Abstract

Aiming at the problem that the main circuit grounding fault in the traction drive system of locomotives and high-speed trains can only be diagnosed under a single operating condition and cannot be warned about early, a mechanism and data-driven real-time evaluation and full operating condition fault warning method for ground insulation degradation is proposed. Firstly, based on the mechanism of grounding faults, the circuit characteristics of the main circuit of the traction transmission system under different grounding fault conditions are analyzed, and mathematical models are established for the detection of various grounding faults and sensor signals under different operating conditions, as well as for evaluating the degree of degradation of grounding faults. Secondly, based on engineering application experience, a feature index set that can accurately classify different types of grounding faults is extracted. Combined with on-site fault case data, a decision tree method is used to establish a classification model between the feature index set and typical grounding fault sources under different operating conditions, which is then converted into a fault diagnosis rule library. Finally, real-time collection of relevant sensor signals, based on the fault diagnosis rule library and the degradation degree evaluation model of grounding faults, enables real-time detection and warning of grounding faults under all operating conditions to ensure train safety and provide key information support for optimal degraded operation in the future. The test result based on controller hardware in the loop shows that the method proposed in this paper can achieve accurate detection and localization of grounding faults under different operating conditions and can provide real-time warning of the severity of grounding faults, which has good engineering application value.

## 1. Introduction

Compared with air and road transportation, rail transport offers advantages such as large capacity, energy efficiency, and punctuality, which have contributed to its rapid development in recent years. Many countries have established a complete railway network, such as Germany’s ICE, France’s TGV, Japan’s Shinkansen, and China’s CRH [1]. By the end of 2024, China’s railway operating mileage will exceed 160,000 km, of which high-speed railways will exceed 46,000 km, accounting for more than two-thirds of the world’s total high-speed railway mileage. As a core component of the high-speed railway system, any minor or potential faults of high-speed trains during operation may trigger a chain reaction and cause accidents or even lead to catastrophic consequences if they cannot be diagnosed and handled in time [2]. Historically, high-speed train safety accidents at home and abroad, such as the ICE high-speed train derailment accident in Germany in 1998 [3] and the “7.23” Ningbo-Wenzhou Electric Multiple Unit (EMU) rear-end collision accident in China in 2011 [4], have caused significant casualties and economic losses.

The traction drive system is the heart of high-speed trains. During the operation of the train, due to cable aging, vibration friction, dust, moisture, and impact from foreign objects, the insulation of electrical components from the ground in the traction drive system will decrease, and eventually a main circuit grounding fault will occur. Single-point grounding can cause the failure of electrical insulation between the main circuit system on the secondary side of the traction transformer and the primary side of the traction transformer; two-point or multi-point grounding will generate a large short-circuit current, causing electrical components to burn. Therefore, it is of great practical significance to study the real-time diagnosis and effective prediction method of the main circuit grounding fault of the traction drive system.

In recent years, many scholars and engineers have conducted extensive and in-depth research on the detection and diagnosis methods of ground faults. In [5], common grounding schemes and protection devices for power systems are introduced in detail. In [6], based on laboratory equipment, the low-frequency coefficients of discrete wavelet transform coefficients and the change rules of load and secondary grounding point are experimentally analyzed for the secondary grounding problem of single-phase traction transformers, which provides a basis for subsequent analysis and location of traction transformer faults. In [7], the shortcomings of the currently commonly used IEC standard A-type (AC side only) differential current protection circuit and grounding detection relay are explained, and the principle and advantages of the B-type (AC/DC) grounding protection device in the variable frequency drive system are analyzed in detail. In [8,9,10], a load-side grounding diagnosis method for variable frequency speed regulation system is proposed. This method requires the addition of a hardware circuit to collect the neutral point-to-ground voltage, and the load-side grounding diagnosis function is realized by analyzing and summarizing the change rules of the neutral point-to-ground voltage when grounding. However, this method requires the addition of hardware and is only applicable to load-side grounding diagnosis. Reference [11] conducted a detailed analysis of the ground detection devices, detection principles, and ground detection methods of three batches of “HX” models. The analysis showed the superiority of the ground detection methods of HXD1 and HXD3 models. Reference [12] gave a detailed introduction to the grounding detection principle and typical faults of the main circuit of the HXD1C locomotive. Reference [13] conducted a comparative analysis based on the equal-division and bias-type voltage-dividing circuits commonly used in the main circuit grounding detection of AC transmission electric locomotives and proposed corresponding fault protection strategies. Reference [14] analyzed the grounding detection principle of the main circuit of the “China Star” EMU, reviewed the on-site fault cases, and discussed the problems existing in the current simple threshold over-limit judgment method. Reference [15] considered the correlation between the measured variables of the traction system, a fault detection method based on canonical correlation analysis (CCA) was proposed, and the residual and fault direction information were used to achieve fault isolation. Reference [16] improved the method of [15] and proposed a CCA method based on feature correlation. The proposed method in [16] was validated by experiments to be superior to the traditional CCA method in terms of FDR and CIR indicators. Reference [17] introduces the basic principles of ground fault diagnosis in the CR400BF EMU traction system, analyzes the characteristics of the ground voltage in the traction system at different ground fault points, and proposes a method for locating and detecting the ground fault point. The effectiveness of the method is validation through actual vehicle tests. References [18,19] respectively explored the grounding detection principles of traction systems in high-speed and urban EMUs, examined the software-based methods for automatically locating grounding points, and carried out experimental validation to support their findings.

The above-mentioned methods do not consider the applicability of fault diagnosis methods under all operating conditions and only perform fault diagnosis based on the signal characteristics under the condition of complete grounding, which cannot achieve the effect of fault warning. Therefore, there are certain limitations in terms of real-time fault detection and ensuring the availability of trains on the way. To this end, this paper deeply analyzes and models the equivalent circuit characteristics under different grounding fault conditions, extracts a strongly correlated feature index set for effective fault classification, and realizes real-time evaluation of the degree of insulation degradation to the ground, providing effective warnings across all operating conditions.

The main innovations of this paper are as follows:

(1) Through in-depth analysis of the fault mechanism, a real-time evaluation method for insulation degradation of traction drive system based on the joint drive of mechanism and data was proposed, and a ground fault degradation degree evaluation model was established.
(2) Combining engineering application experience and field fault case data, a classification model of characteristic index sets and typical ground fault sources was constructed based on the decision tree method and converted into a fault diagnosis rule library.
(3) Based on the fault diagnosis rule base and the grounding fault degradation degree assessment model, real-time detection and early warning of multiple operating conditions of grounding faults are realized, providing key information support for the subsequent optimal degradation operation.

The rest of the paper is organized as follows: Section 2 introduces the main circuit operating conditions and the grounding characteristics analysis under different operating conditions. Section 3 proposes a real-time diagnosis method. Section 4 describes the experimental process and compares the experimental results. Section 5 concludes this article.

## 2. Introduction to Traction System Operating Conditions and Analysis of Grounding Circuit Characteristics

### 2.1. Introduction to the Main Circuit Principle of Traction System

**Figure 1:**
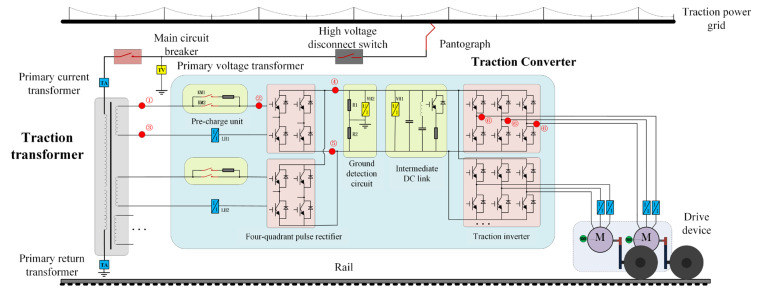
Schematic diagram of main circuit of traction system.

The main circuit of a typical traction system for a locomotive and EMU is shown in Figure 1. It mainly consists of three parts: a traction transformer, a traction converter (including a charging circuit, a four-quadrant rectifier, a ground detection circuit, an intermediate DC link, a traction inverter, etc.), and a traction motor. Single-phase AC 25 kV power enters the vehicle body through the pantograph, the main circuit breaker VCB, and the primary side of the traction transformer. The secondary winding of the traction transformer provides single-phase AC power to the converter circuit. The AC power is converted into DC power by the four-quadrant rectifier. After filtering by the intermediate DC link, it is converted into three-phase AC power with variable frequency and amplitude by the inverter to drive the traction motor, thereby controlling the locomotive to move forward at different speeds and traction forces.

### 2.2. Traction System Operating Conditions and Typical Grounding Types

According to the different operating conditions of the main circuit-related components of the traction drive system, the system working process can be divided into five operating conditions, *C*_1_–*C*_5_, as shown in Table 1. The startup process or fault re-entry process of the locomotive and EMU traction system generally goes through conditions *C*_1_–*C*_5_ or one or several of them in sequence. The common grounding fault types of the main circuit are summarized in Table 2. It should be noted that since fault types *F*_1_ and *F*_2_ are equipotential points in conditions *C*_3_–*C*_5_, we usually combine them into the same fault type in engineering.

### 2.3. Analysis of Circuit Characteristics Under Different Grounding Conditions in Various Operating Conditions

The main circuit grounding detection circuit and common grounding points of the traction system are shown in Figure 1. The primary voltage transformer TV collects the primary voltage of the traction transformer, the voltage sensors VH1 and VH2 are used to collect the intermediate DC voltage and grounding detection voltage of the main circuit, respectively, and the current sensor LH1 is used to collect the four-quadrant input current. The markings ①–⑥ in the figure are common main circuit grounding types, and their detailed descriptions are shown in Table 2.

**Figure 2:**
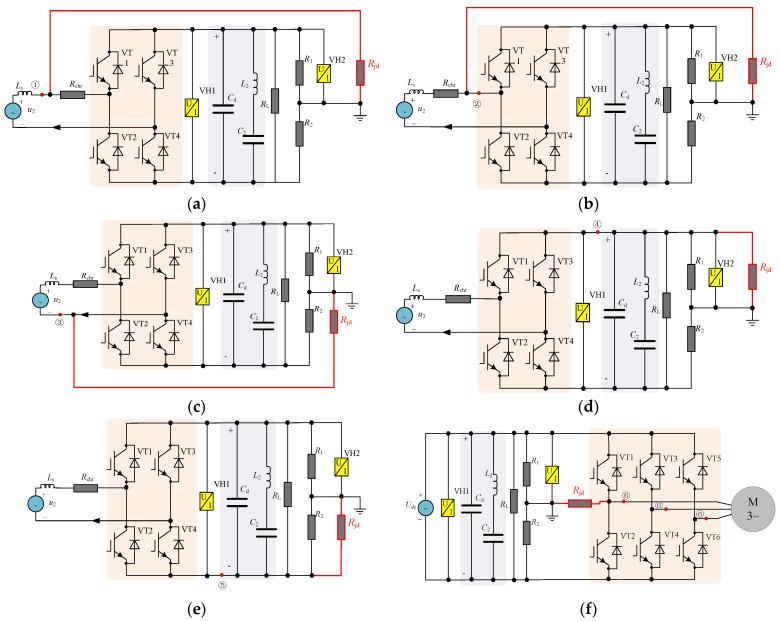
Equivalent circuits of different grounding types. (**a**) Positive terminal of the secondary winding grounding. (**b**) Positive terminal of the four-quadrant input side grounding. (**c**) Negative terminal of the four-quadrant input side grounding. (**d**) Positive busbar grounding. (**e**) Negative busbar grounding. (**f**) Inverter output side grounding.

Figure 2 shows the equivalent circuits of different grounding types. Different operating conditions can be changed by adjusting the value of charging resistance *R*_chr_. In working condition *C*_1_, *R*_chr_ is set to infinity; in operating condition *C*_2_, it is set to the actual charging resistance value; and in operating conditions *C*_3_–*C*_5_, *R*_chr_ is set to 0. In Figure 2, *R*_jd_ is the value of grounding insulation resistance. Under normal circumstances, the value of the system connection insulation resistance *R*_jd_ > 2 MΩ. When there is insulation degradation, its resistance value gradually decreases, and when there is complete grounding, its value is close to 0. The following is a detailed analysis of the fault characteristics of various fault points under different operating conditions.

#### 2.3.1. Positive Terminal of the Secondary Winding Grounding

The equivalent circuit of the secondary winding positive terminal grounding is shown in Figure 2a. From the equivalent circuit analysis, when the secondary winding positive terminal is grounded, the change law of the ground detection voltage sensor VH2 sampling value *U_jd_* under different operating conditions can be calculated based on Equations (1)–(4):

(1) $$U_{jd1}^{C1}=\left\{\begin{array}{cc} -\frac{R_{1}+R_{jd}}{R_{1}+2R_{jd}}\cdot u_{2} & ,u_{2}<-0.5\cdot U_{dc}+e_{1}\\ 0.5\cdot U_{dc} & ,\left|u_{2}\right|\leq 0.5\cdot U_{dc}+e_{1}\\ U_{dc}-\frac{R_{1}+R_{jd}}{R_{1}+2R_{jd}}\cdot u_{2}\; & ,u_{2}>0.5\cdot U_{dc}+e_{1} \end{array}\right.$$

(2) $$U_{jd1}^{C2}=\left\{\begin{array}{cc} \frac{R_{jd}\cdot U_{dc}-R_{1}\cdot R_{chr}\cdot i_{qc}}{R_{1}+2R_{jd}} & ,i_{qc}>e_{2}\\ 0.5\cdot U_{dc} & ,\left|i_{qc}\right|<e_{2}\\ \frac{(R_{1}+R_{jd})\cdot U_{dc}-R_{1}\cdot R_{chr}\cdot i_{qc}}{R_{1}+2R_{jd}} & ,i_{qc}<-e_{2} \end{array}\right.$$

(3) $$U_{jd1}^{C3}=\left\{\begin{array}{cc} \frac{R_{jd}\cdot U_{dc}}{R_{1}+2R_{jd}} & ,i_{qc}>e_{2}\\ -\frac{R_{1}+R_{jd}}{R_{1}+2R_{jd}}\cdot u_{2} & ,\left|i_{qc}\right|\leq e_{2}\&u_{2}<-0.5U_{dc}+e_{1}\\ 0.5\cdot U_{dc} & ,\left|i_{qc}\right|\leq e_{2}\&\left|u_{2}\right|\leq 0.5U_{dc}+e_{1}\\ U_{dc}-\frac{R_{1}+R_{jd}}{R_{1}+2R_{jd}}\cdot u_{2}\; & ,\left|i_{qc}\right|\leq e_{2}\&u_{2}>0.5U_{dc}+e_{1}\\ \frac{(R_{1}+R_{jd})\cdot U_{dc}}{R_{1}+2R_{jd}} & ,i_{qc}<-e_{2} \end{array}\right.$$

(4) $$U_{jd1}^{C4,5}=\left\{\begin{array}{cc} \frac{R_{jd}\cdot U_{dc}}{R_{1}+2R_{jd}} & ,S_{A}=1\\ \frac{(R_{1}+R_{jd})\cdot U_{dc}}{R_{1}+2R_{jd}} & ,S_{A}=0 \end{array}\right.$$

In the formula, *u*_2_ is the sampling value of the high-voltage voltage transformer TV converted to the secondary winding, *U_dc_* is the sampling value of the voltage sensor VH1, and *i_qc_* is the sampling value of the current sensor LH1; *S_A_* is related to the opening state of the A-phase bridge arm (VT1 and VT2) of the four-quadrant module, and *S_A_* is 1 when VT1 is on and *S_A_* is 0 when VT2 is on; *R_jd_* is the insulation resistance to the ground; and is the reconstructed value of the grounding detection voltage *U_jd_* when a secondary winding positive terminal grounding (*F*_1_) fault occurs under the operating condition *C_i_* (*i* = 1, …, 5).

#### 2.3.2. Positive Terminal of the Four-Quadrant Input Side Grounding

From Figure 2b and the analysis in Section 2.3.1, when the positive terminal of the four-quadrant input side is grounded under operating conditions *C*_3_–*C*_5_, the calculation formula is consistent with Formulas (3) and (4). Under operating conditions *C*_1_ and *C*_2_, the calculation formula is shown in Formulas (5) and (6), respectively.

(5) $$U_{jd2}^{C1}=0.5U_{dc}$$

(6) $$U_{jd2}^{C2}=\left\{\begin{array}{cc} \frac{R_{jd}\cdot U_{dc}}{R_{1}+2R_{jd}} & ,i_{qc}>e_{2}\\ 0.5\cdot U_{dc} & ,\left|i_{qc}\right|<e_{2}\\ \frac{(R_{1}+R_{jd})\cdot U_{dc}}{R_{1}+2R_{jd}} & ,i_{qc}<-e_{2} \end{array}\right.$$

#### 2.3.3. Negative Terminal of the Four-Quadrant Input Side Grounding

When the negative terminal of the four-quadrant input side is grounded, it can be seen from its equivalent circuit Figure 2c that the change rule of its ground detection voltage VH2 can be calculated based on Equations (7)–(9):

(7) $$U_{jd3}^{C1}=0.5U_{dc}$$

(8) $$U_{jd3}^{C2,3}=\left\{\begin{array}{cc} \frac{(R_{1}+R_{jd})\cdot U_{dc}}{R_{1}+2R_{jd}} & ,i_{qc}>e_{2}\\ \;U_{dc}+\frac{R_{1}+R_{jd}}{R_{1}+2R_{jd}}\cdot u_{2} & ,\left|i_{qc}\right|\leq e_{2}\&u_{2}<-0.5U_{dc}+e_{1}\\ 0.5\cdot U_{dc} & ,\left|i_{qc}\right|\leq e_{2}\&\left|u_{2}\right|\leq 0.5U_{dc}+e_{1}\\ \frac{R_{1}+R_{jd}}{R_{1}+2R_{jd}}\cdot u_{2} & ,\left|i_{qc}\right|\leq e_{2}\&u_{2}>0.5U_{dc}+e_{1}\\ \frac{R_{jd}\cdot U_{dc}}{R_{1}+2R_{jd}} & ,i_{qc}<-e_{2} \end{array}\right.$$

(9) $$U_{jd3}^{C4,5}=\left\{\begin{array}{cc} \frac{R_{jd}\cdot U_{dc}}{R_{1}+2R_{jd}} & ,S_{B}=1\\ \frac{(R_{1}+R_{jd})\cdot U_{dc}}{R_{1}+2R_{jd}} & ,S_{B}=0 \end{array}\right.$$

In the formula, *S*_B_ is related to the opening state of the B-phase bridge arm (VT3 and VT4) of the four-quadrant module, and *S_B_* is 1 when VT3 is on and 0 when VT4 is on; $U_{jd3}^{Ci}$ is the reconstructed value of the ground detection voltage *U*_jd_ when a four-quadrant input side negative terminal ground fault (*F*_3_) occurs under the operating condition *C_i_* (*i* = 1, …, 5).

#### 2.3.4. Intermediate DC Link Grounding

When there is intermediate DC link grounding, it can be seen from the analysis of Figure 2d,e that the calculated value of the grounding detection voltage is only related to the intermediate voltage sampling value and the insulation resistance to ground *R_jd_*. When the positive busbar is grounded, and the negative busbar is grounded, the calculation formulas are shown in Formulas (10) and (11), respectively.

(10) $$U_{jd4}^{Ci}=\frac{R_{jd}\cdot U_{dc}}{R_{1}+2R_{jd}}$$

(11) $$U_{jd5}^{Ci}=\frac{(R_{1}+R_{jd})\cdot U_{dc}}{R_{1}+2R_{jd}}$$

In the formula, $U_{jd4}^{Ci}$ and $U_{jd5}^{Ci}$ are the reconstructed values of the grounding detection voltage *U_jd_* when the positive busbar grounding (*F*_4_) and negative busbar grounding (*F*_5_) faults occur under the operating condition *C_i_* (*i* = 1, …, 5).

#### 2.3.5. Inverter Output Side Grounding

When there is grounding on the inverter output side, the grounding detection value of the system in operating conditions *C*_1_–*C*_4_ is no different from the normal situation. Based on the analysis of Figure 2f, when entering operating condition *C*_5_, the sampling value of the grounding detection voltage sensor VH2 will change between 0 and *U_s_*_1_ with the inverter switch action state (the change frequency is equal to the switching frequency when the inverter module is working). Taking the U phase grounding on the inverter output side as an example, its change rule can be calculated using Formula (12):

(12) $$U_{jd6}^{C5}=\left\{\begin{array}{cc} \frac{R_{jd}\cdot U_{dc}}{R_{1}+2R_{jd}} & ,S_{U}=1\\ \frac{(R_{1}+R_{jd})\cdot U_{dc}}{R_{1}+2R_{jd}} & ,S_{U}=0 \end{array}\right.$$

In the formula, *S_U_* is related to the opening state of the U-phase bridge arm (VT1 and VT2) of the inverter module; when VT1 is on, *S_U_* is 1, and when VT2 is on, *S_U_* is 0. The grounding conditions of the V-phase and W-phase are similar to those of the U-phase.

## 3. Real-Time Diagnosis of Ground Faults

### 3.1. Fault Detection

As can be seen from Section 2.3.1, the value of ground detection voltage *U*_jd_ under different fault conditions is not the same, so fault detection is performed by designing the residual *r* related to it. The designed residual *r* is shown in Formula (13). During normal operation, if the system has no ground fault (this paper takes *R_jd_* ≥ 20 kΩ), the residual r satisfies $r\sim N(\mu_{0},\sigma_{0}^{2(Ci)})$.

Among them, $\mu_{0}=0$ is the mean of *r*, $\sigma_{0}^{2(Ci)}$ is the variance of the residual of each operating condition under the condition of no fault, and its value can be learned through normal historical data.

(13) $$r=u_{e}=U_{jd}-0.5\cdot U_{dc}$$

In the formula, *u_e_* is the ground fault detection quantity.

Let $R=\{r^{\left(1\right)},r^{\left(2\right)},\cdots ,r^{\left(N\right)}\}$ be the periodic sampling value of *r*, where *N* is the number of samples in the sliding period window. The detection statistic *T*^2^ is defined as shown in Formula (14).

(14) $$T^{2}=\frac{\sum_{i=1}^{N}{\left(r^{\left(i\right)}\right)}^{2}}{\sigma_{0}^{2(Ci)}}$$

In the formula, *T*^2^ satisfies the $\chi^{2}$ standard distribution with *N* − 1 degrees of freedom, and $p(T^{2}>\chi_{\alpha }^{2}|H_{0})=\alpha$; $p(T^{2}>\chi_{\alpha }^{2}|H_{0})$ represents the probability that the detection quantity *T*^2^ is greater than $\chi_{\alpha }^{2}$ under the fault-free hypothesis *H*_0_.

This paper adopts this method for fault detection, and the threshold is obtained by approximating the chi-square distribution, that is,

(15) $$T_{\alpha }=\chi_{\alpha }^{2}(N)$$

In the formula, $T_{\alpha }$ represents the threshold, $\chi_{\alpha }^{2}(N)$ represents the chi-square distribution with *N* degrees of freedom, and α represents the confidence level, which is usually understood as the probability of allowing false detection.

### 3.2. Fault Tracing Decision Tree Modeling

Next, by analyzing the variation law of residual *r* under different grounding faults in various operating conditions, the relevant fault characteristic index is constructed, and the decision tree classification method is used to model the fault classification, so as to achieve accurate classification of different grounding faults.

From Formula (13) and the analysis in Section 2.3.1, the residual *r* is mainly related to *u*_2_ and *U_jd_*, and the degree of its deviation from the normal value is strongly related to the degree of insulation degradation, that is, the value of *R_jd_*. When the system is in operating condition *C*_1_–*C*_3_, *U_jd_* is a piecewise function related to *i_qc_*, *U_dc_*, and *u*_2_, while when the system is in operating condition *C*_4_–*C*_5_ and *F*_1_, *F*_2_, and *F*_5_ appear, *U_jd_* is a high-frequency square wave signal strongly related to the IGBT switching state.

Based on the above mechanism analysis and application experience, the mean and variance of the residual *r* in the periodic sliding window, the product accumulation value of *r* and *u*_2_, and the maximum and minimum values of *r* are selected as five characteristic indicators, which are respectively recorded as *J*_1_–*J*_5_, and their definitions are shown in Formulas (16)–(20).

(16) $$J_{1}(k)=\frac{1}{N}\sum_{i=1}^{N}r(k-i+1)$$

(17) $$J_{2}(k)=\frac{1}{N-1}\sum_{i=1}^{N}{\left[r(k-i+1)-J_{1}(k)\right]}^{2}$$

(18) $$J_{3}(k)=\frac{1}{N}\sum_{i=1}^{N}\left[r(k-i+1)\cdot u_{2}(k-i+1)\right]$$

(19) $$J_{4}(k)=\max(\{r(k-i+1)|i=1,\cdots ,N\})$$

(20) $$J_{5}(k)=\min(\{r(k-i+1)|i=1,\cdots ,N\})$$

**Figure 3:**
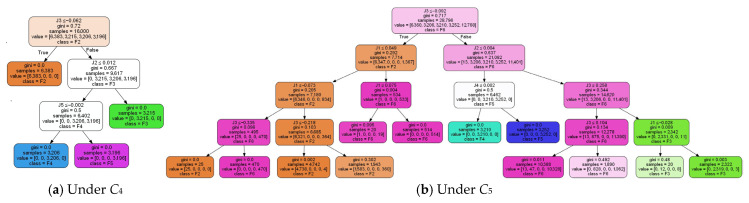
Decision tree diagnosis model for main circuit grounding under multiple operating conditions.

Based on the field fault case data, the characteristic indicators are calculated according to Equations (16)–(20) to form a model data sample set and a test set. After randomly sorting the sample data, 80% of them are selected for training, and the remaining 20% are used for model classification accuracy calculation. The multi-operating condition decision tree diagnosis model of the main circuit grounding fault in the traction drive system is obtained as shown in Figure 3. After the decision tree diagnosis model is established, the IF-ELSE rule is used to convert it into a diagnostic rule base that can be implemented online for use in the subsequent fault decision process.

### 3.3. Failure Decision

**Figure 4:**
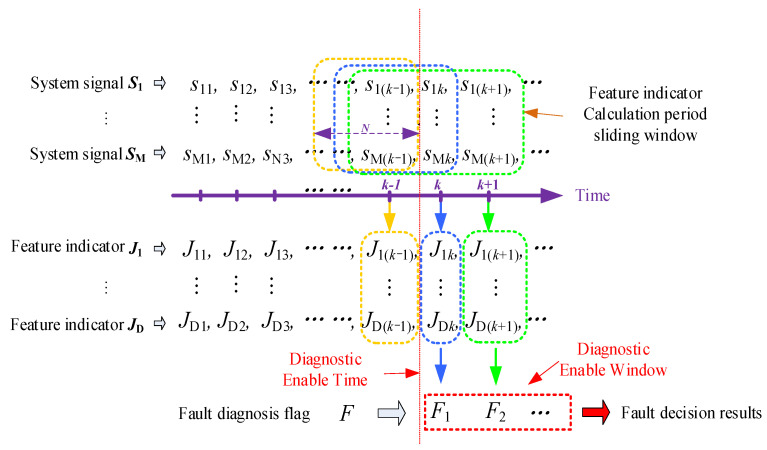
Schematic diagram of signal timing windowing processing.

After the offline design of the diagnostic rule base, the relevant sensor signals can be collected online, their characteristic indicators can be calculated in real time, and rule matching can be performed based on the above diagnostic rule base. To ensure the reliability of the classification results, this paper designs a diagnostic enable window (as shown in Figure 4) to effectively avoid fault misdiagnosis caused by dynamic changes in the system during the operating condition switching process. On this basis, a comprehensive decision is made through the fault diagnosis flags in the diagnosis enable window, and the center of gravity method is used to achieve the final fault tracing decision output.

### 3.4. Fault Warning

The purpose of fault warning is to monitor the degradation state in real time based on the system-related health status characterization quantity, so that the system can timely predict its abnormal condition before the actual fault occurs in order to take scientific and reasonable driver disposal or maintenance plans, thereby improving the safety, reliability, and availability of the traction drive system.

The insulation resistance *R_jd_* to ground in the equivalent circuit of different grounding types shown in Figure 2 is directly related to the severity of grounding and can be used as an indirect characterization parameter of the system degradation state. Based on this parameter, the system health status is evaluated. Therefore, to achieve ground fault warning, it is necessary to estimate *R_jd_* at different ground faults.

From the analysis in Section 2.3.1, *R_jd_* is strongly correlated with the ground detection voltage value *U_jd_*, the intermediate voltage *U_dc_*, and the secondary voltage *u*_2_, and the relationship is different under different operating conditions. Based on the relevant relationship in Section 2.3.1, the estimated value ${\hat{R}}_{jd}$ of the insulation resistance to ground of the ground fault *F*_1_–*F*_6_ under different operating conditions can be derived. The calculation formula is shown in Table 3.

In the table, the calculation formulas for ${\hat{R}}_{jd}^{\left(1\right)}$–${\hat{R}}_{jd}^{\left(6\right)}$ are shown in Formulas (21)–(26) respectively.

(21) $${\hat{R}}_{jd}^{\left(1\right)}=\frac{-R_{1}(u_{2}+U_{jd})}{u_{2}+2U_{jd}}$$

(22) $${\hat{R}}_{jd}^{\left(2\right)}=\frac{-R_{1}(u_{2}+U_{jd}-U_{dc})}{u_{2}+2(U_{jd}-U_{dc})}$$

(23) $${\hat{R}}_{jd}^{\left(3\right)}=\frac{R_{1}\cdot R_{chr}\cdot i_{qc}+R_{1}\cdot U_{jd}}{U_{dc}-2U_{jd}}$$

(24) $${\hat{R}}_{jd}^{\left(4\right)}=\frac{R_{1}\cdot R_{chr}\cdot i_{qc}+R_{1}\cdot (U_{jd}-U_{dc})}{U_{dc}-2U_{jd}}$$

(25) $${\hat{R}}_{jd}^{\left(5\right)}=\frac{R_{1}\cdot U_{jd}}{U_{dc}-2U_{jd}}$$

(26) $${\hat{R}}_{jd}^{\left(6\right)}=\frac{R_{1}\cdot (U_{jd}-U_{dc})}{U_{dc}-2U_{jd}}$$

After estimating the insulation resistance to ground, this paper divides its health status into four different levels according to its size: A (normal), B (weakened), C (abnormal), and D (fault). The insulation resistance value ranges corresponding to different health states are shown in Table 4.

**Figure 5:**
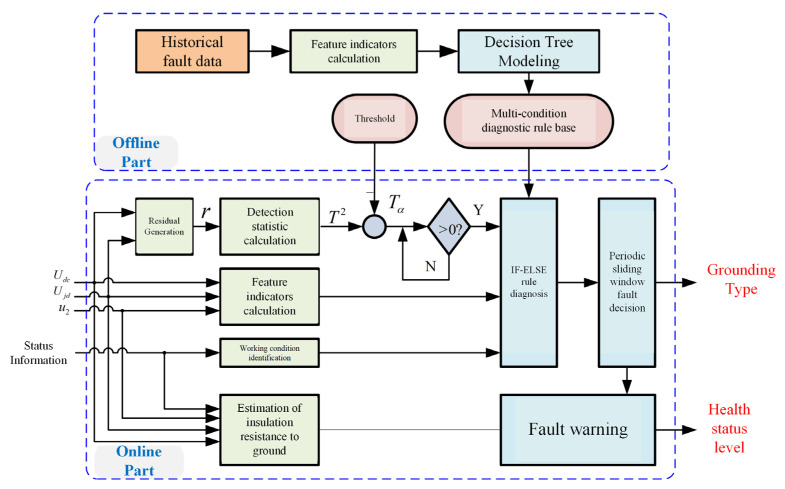
Principal block diagram of real-time evaluation and real-time warning algorithm for insulation degradation of traction drive system to ground.

The entire ground fault real-time diagnosis and fault-tolerant operation optimization control principal block diagram is shown in Figure 5.

## 4. Experimental Validation

**Figure 6:**
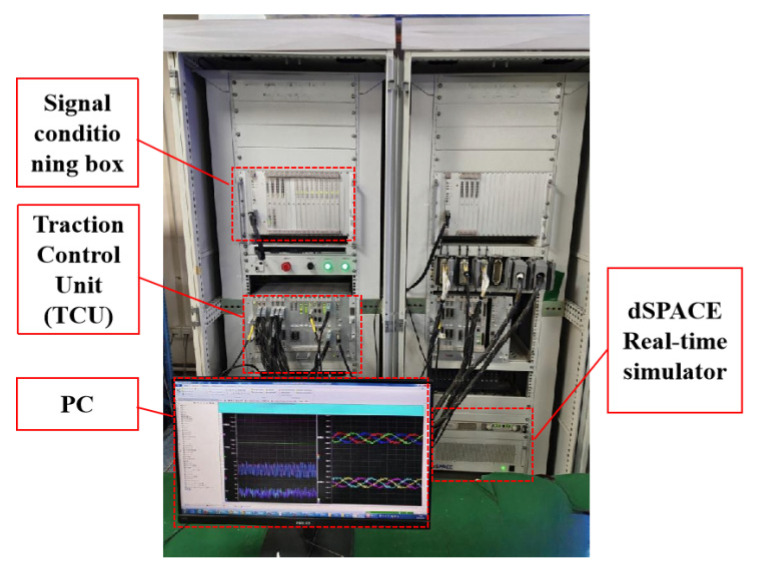
Test platform for traction drive system fault testing and algorithm validation.

Based on the relevant system parameters of a certain type of train traction system, a test platform for traction drive system fault testing based on controller-in-the-loop is built as shown in Figure 6 to test and validate the proposed algorithm. The test platform consists of a signal conditioning chassis, a real traction control unit (TCU), a real-time simulator (dSPACE), etc. Among them, the signal conditioning chassis realizes the signal conversion between the chassis under test (TCU) and the real-time simulator; the real-time simulator performs real-time calculations of the model with fault injection capabilities, outputs real-time external signals, and enables main circuit modeling, model downloading, and real-time data monitoring through a PC. This paper uses the real-time simulator to simulate the main circuit grounding fault conditions of various severity levels and implement the fault diagnosis algorithm in the chassis under test (TCU).

**Figure 7:**
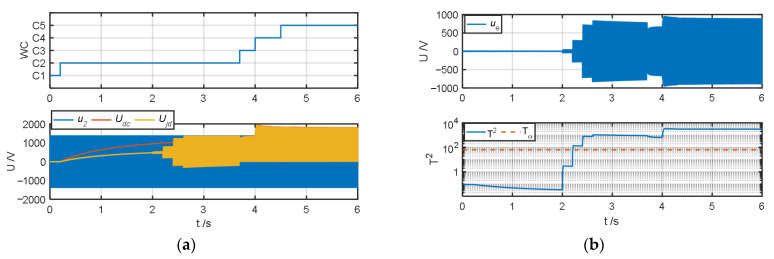

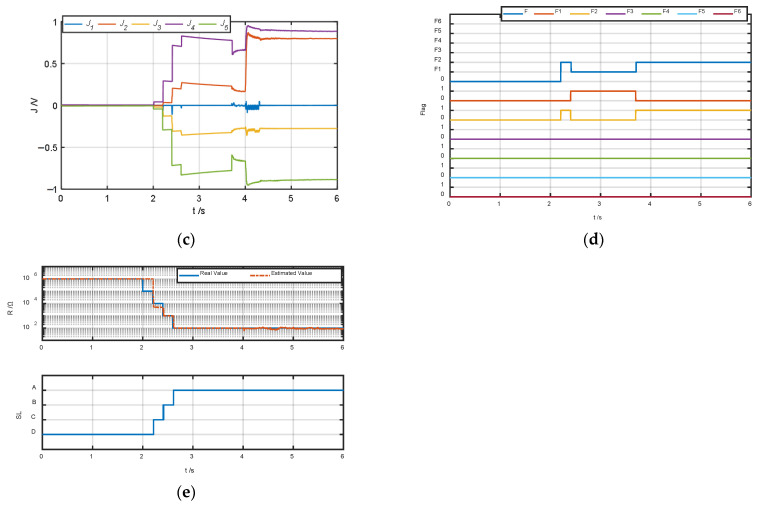
Fault warning algorithm test results when fault type is *F*_1_. (**a**) Correlation sampling status signal. (**b**) Fault detection results. (**c**) Related fault characteristic indicators. (**d**) Fault decision results. (**e**) Status assessment results.

**Figure 8:**
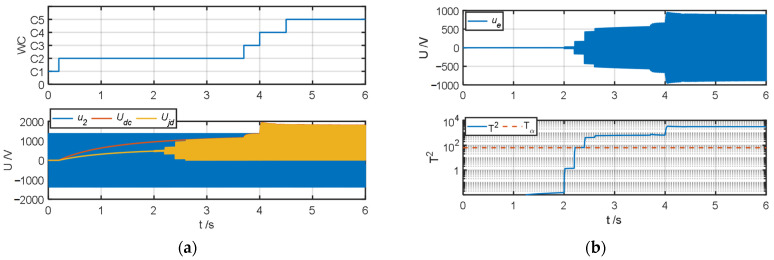

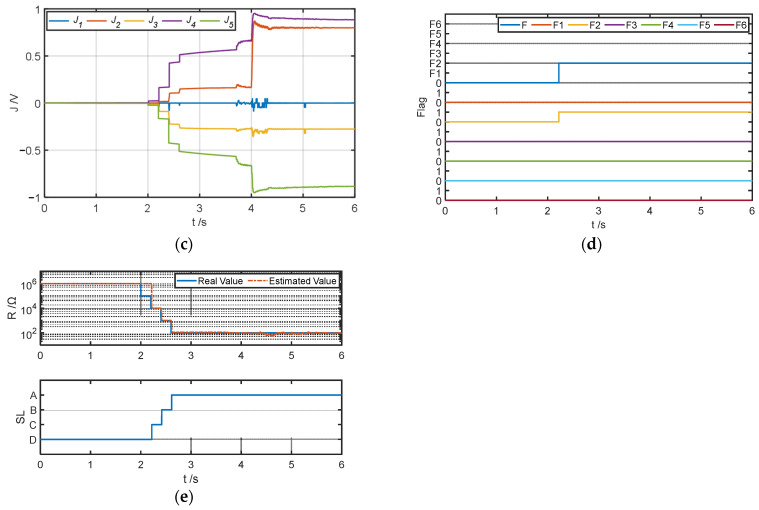
Fault warning algorithm test results when fault type is *F*_2_. (**a**) Correlation sampling status signal. (**b**) Fault detection results. (**c**) Related fault characteristic indicators. (**d**) Fault decision results. (**e**) Status assessment results.

**Figure 9:**
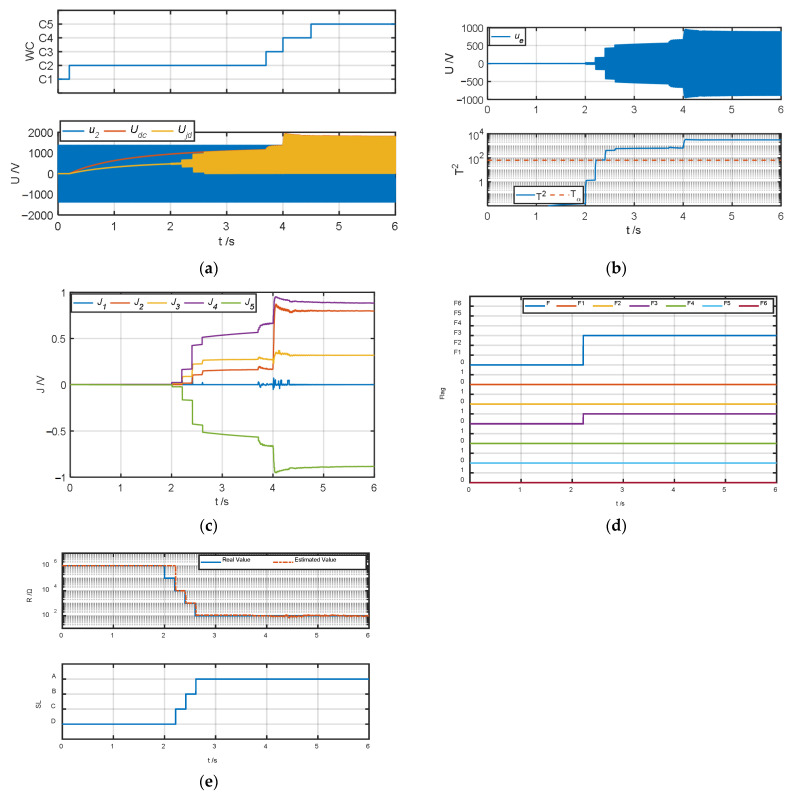
Fault warning algorithm test results when fault type is *F*_3_. (**a**) Correlation sampling status signal. (**b**) Fault detection results. (**c**) Related fault characteristic indicators. (**d**) Fault decision results. (**e**) Status assessment results.

**Figure 10:**
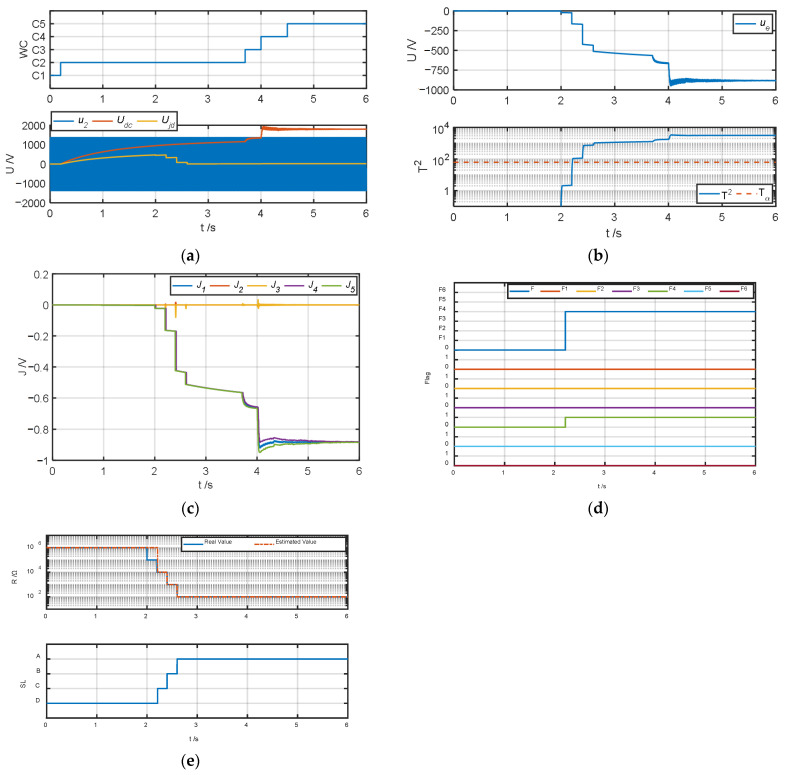
Fault warning algorithm test results when fault type is *F*_4_. (**a**) Correlation sampling status signal. (**b**) Fault detection results. (**c**) Related fault characteristic indicators. (**d**) Fault decision results. (**e**) Status assessment results.

**Figure 11:**
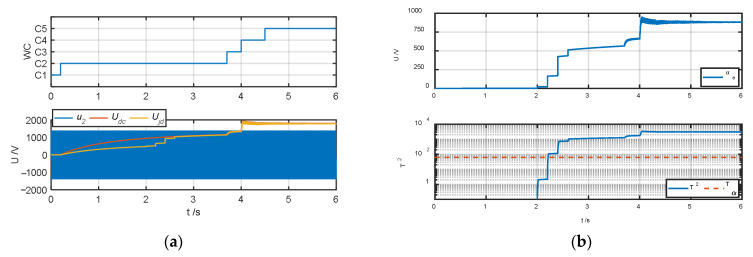

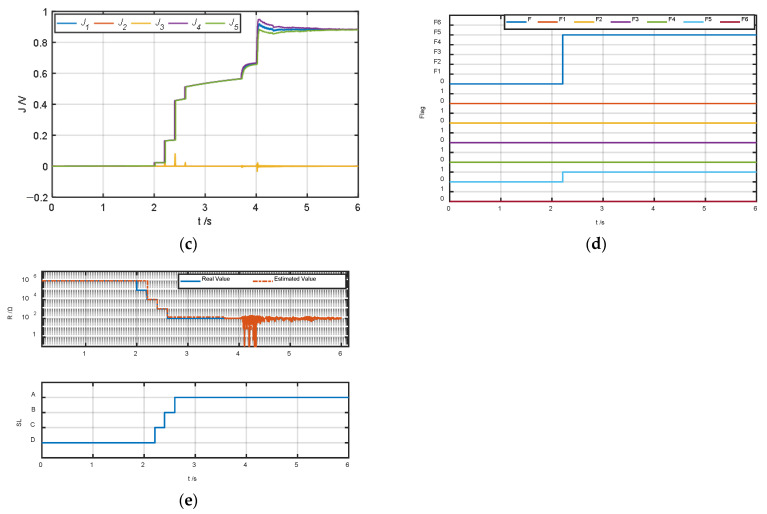
Fault warning algorithm test results when fault type is *F*_5_. (**a**) Correlation sampling status signal. (**b**) Fault detection results. (**c**) Related fault characteristic indicators. (**d**) Fault decision results. (**e**) Status assessment results.

**Figure 12:**
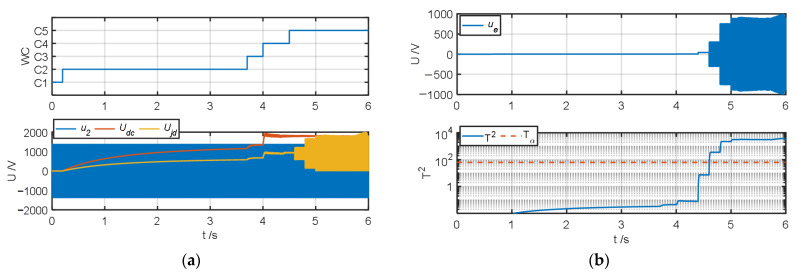

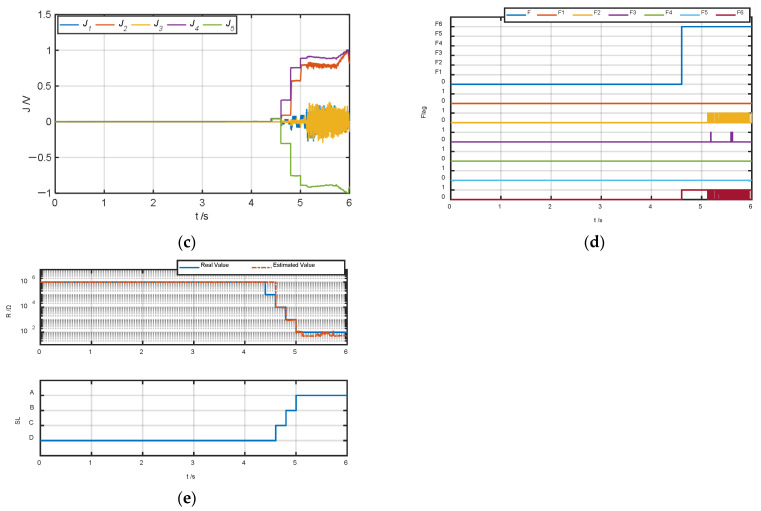
Fault warning algorithm test results when fault type is *F*_6_. (**a**) Correlation sampling status signal. (**b**) Fault detection results. (**c**) Related fault characteristic indicators. (**d**) Fault decision results. (**e**) Status assessment results.

The authors tested and validated the proposed algorithm by changing the insulation resistance to ground to simulate different degrees of ground faults in the system. Because all faults except fault type *F*_6_ can be diagnosed in operating conditions *C*_2_–*C*_4_, the authors set the default insulation resistance to ground to 1 MΩ. When testing fault types *F*_1_–*F*_5_, the authors simulated ground faults of different severities at t = 2 s, 2.2 s, 2.4 s, and 2.6 s and changed the insulation resistance *R_jd_* to 100 kΩ, 10 kΩ, 1 kΩ, and 100 Ω, respectively; when the fault type was *F*_6_, the authors simulated ground faults of different severities at t = 4.4 s, 4.6 s, 4.8 s, and 5 s and obtained the test results of the fault warning algorithm as shown in Figure 7, Figure 8, Figure 9, Figure 10, Figure 11 and Figure 12.

Figure 7 and Figure 8 show the fault diagnosis results when the fault types are the positive terminal of the secondary winding grounded (*F*_1_) and the positive terminal of the four-quadrant input side grounded (*F*_2_). As shown in Figure 7a and Figure 8a, the system operates in *C*_1_–*C*_5_ conditions in sequence. When the system insulation resistance to ground *R_jd_* changes from 1 MΩ to 100 kΩ at t = 2 s, the system does not detect the ground fault because *R_jd_* does not meet the condition of less than 20 kΩ. After t = 2.2 s, when the insulation resistance to ground meets the condition of less than 20 kΩ, it can be seen from Figure 7b and Figure 8b that the amplitude of the ground fault detection quantity *u*_e_ increases significantly, and its corresponding detection statistic *T*^2^ quickly exceeds the detection threshold. When *F*_1_ and *F*_2_ grounding occurs, the change trends of their fault characteristic indicators *J*_1_–*J*_5_ are the same, the amplitudes of *J*_2_ and *J*_4_ increase positively, the amplitudes of *J*_3_ and *J*_5_ increase negatively, and *J*_1_ has basically no obvious change, as shown in Figure 7c and Figure 8c. From the analysis of Figure 7d, under the *C*_2_ operating condition, when the real fault type is *F*_1_ and the insulation resistance is 10 kΩ, the system misjudges it as *F*_2_ because the fault characteristic indicators corresponding to *F*_1_ and *F*_2_ are close. However, *F*_1_ and *F*_2_ are generally regarded as the same fault type in engineering, so the misjudgment here will not cause any impact; when the insulation resistance continues to drop to 1 kΩ or below, the system can normally distinguish between *F*_1_ and *F*_2_. At the same time, from the analysis of Figure 8d, when the fault type is *F*_2_, the real fault type can be accurately detected. From Figure 7e and Figure 8e, when the fault types are *F*_1_ and *F*_2_, effective estimation of the insulation resistance to ground and assessment of the degradation state can be achieved.

The fault diagnosis result when the fault type is the grounding of the negative terminal of the four-quadrant input side (*F*_3_) is shown in Figure 9. Comparing Figure 9b with Figure 8b, when the fault type is *F*_3_, the amplitude of the ground fault detection quantity is not significantly different from that of *F*_2_. However, it can be seen from Figure 9c that when the fault is *F*_3_, the amplitude of the characteristic index *J*_3_ increases significantly in a positive direction, which is significantly different from that of *F*_2_. As shown in Figure 9d, the system correctly diagnoses the fault type *F*_3_ in operating condition *C*_2_ and its subsequent operating conditions.

Figure 10 and Figure 11 are the fault diagnosis results when the fault types are positive busbar grounding (*F*_4_) and negative busbar grounding (*F*_5_), respectively. It can be seen from the corresponding Figure 10a,b and Figure 11a,b that the ground detection voltage value *U_jd_* and the ground fault detection value *U_e_* in these two types of fault conditions are monotonically changing. When the fault type is *F*_4_, its *U_jd_* gradually approaches 0 as the insulation to the ground decreases, and *u*_e_ increases negatively, while when the fault type is *F*_5_, its *U_jd_* gradually approaches the intermediate voltage *U_dc_* as the insulation to the ground decreases, and *u_e_* increases positively. When the fault type is *F*_4_, the corresponding fault characteristic indicators except *J*_3_ increase negatively, while when the fault type is *F*_5_, they increase positively. From Figure 10d and Figure 11d, in both fault conditions, the system correctly diagnoses the corresponding fault type and realizes the effective evaluation of the real insulation state to the ground.

Figure 12 shows the fault diagnosis result when the inverter output side is grounded (*F*_6_). It can be seen from Figure 12a,b that after the fault, the detection voltage value *U_jd_* and the ground fault detection value *U_e_* are both square wave signals with a mean of 0 and a gradually increasing variance; after the fault, the fault characteristic indicators *J*_2_ and *J*_4_ are positively biased, *J*_5_ is negatively biased, and *J*_1_ and *J*_3_ fluctuate rapidly around zero, as shown in Figure 12c. Due to the fluctuation of *J*_1_ and *J*_3_, the fault diagnosis result marks *F*_2_ and *F*_3_ appear as short-term valid marks, but because the proposed algorithm uses the centroid method to make diagnostic decisions, and because *F*_6_ is the longest valid number of times within the cycle window time, the system diagnostic decision also calculates the true fault type and insulation state level.

## 5. Conclusions

This article presents a novel real-time evaluation and fault warning method for ground insulation degradation in the traction drive systems of locomotives and high-speed trains. The proposed method integrates both mechanistic understanding and data-driven approaches to address the critical challenge of early warning and real-time monitoring of grounding faults under multiple operating conditions. The validation result demonstrates the method’s effectiveness in accurately detecting and evaluating various types of grounding faults (*F*1–*F*6) across different operational scenarios. It can be quickly deployed in vehicles based on existing controller hardware, with clear physical concepts, low computational complexity, and easy engineering promotion and application advantages.

In the future, based on the proposed ground fault early warning algorithm, the impact of ground faults of different types and severity on the system will be deeply analyzed, and the differentiated protection strategy of the traction drive system after the fault will be studied to achieve the optimal degraded operation of the train.

## Figures and Tables

**Table 1:** Traction drive system operating conditions description.

Operating Conditions	Operating Conditions Description
C1	The early stage of charging is from the closing of the main circuit breaker to the closing of the charging contactor KM1. This condition is a transient condition and is used to connect the high-voltage power supply.
C2	Pre-charging condition: from the closing of charging contactor KM1 to the closing of shorting contactor KM2, this condition is a transient condition, mainly to reduce the impact current when closing shorting contactor KM2
C3	Short-circuit condition: The short-circuit contactor KM2 is closed, and the four quadrants and the inverter are not running. This condition is a transient condition and is usually experienced when starting or during fault re-entry.
C4	Four-quadrant operating condition: The short-circuit contactor KM2 is closed, there is four-quadrant operation, and the inverter is not running. This condition is usually experienced when starting or coasting or during fault re-entry.
C5	Inverter operation condition: The short-circuit contactor KM2 is closed, and the four quadrants and inverter are all running. This condition is a stable condition, and the train is in this condition during normal traction operation.

**Table 2:** Typical main circuit grounding fault types in traction systems.

Fault Type	Fault Description	Corresponding Markings in Figure 1
*F*_1_	Positive terminal of the secondary winding grounding	Marking point ①
*F*_2_	Positive terminal of the four-quadrant input side grounding	Marking point ②
*F*_3_	Negative terminal of the four-quadrant input side grounding	Marking point ③
*F*_4_	Positive busbar grounding	Marking point ④
*F*_5_	Negative busbar grounding	Marking point ⑤
*F*_6_	Inverter output side grounding	Marking point ⑥

**Table 3:** Estimation of grounding insulation resistance.

Fault Type	Calculation	Operating Conditions
*F*_1_	$\left\{\begin{array}{l} {\hat{R}}_{jd}^{\left(1\right)},u_{2}<-0.5\cdot U_{dc}+e_{1}\\ {\hat{R}}_{jd}^{\left(2\right)},u_{2}>0.5\cdot U_{dc}+e_{1} \end{array}\right.$	*C*_1_
	$\left\{\begin{array}{l} {\hat{R}}_{jd}^{\left(3\right)},i_{qc}>e_{2}\\ \;{\hat{R}}_{jd}^{\left(4\right)},i_{qc}<-e_{2} \end{array}\right.$	*C*_2_
	$\left\{\begin{array}{l} {\hat{R}}_{jd}^{\left(5\right)},i_{qc}>e_{2}\\ \;{\hat{R}}_{jd}^{\left(6\right)},i_{qc}<-e_{2} \end{array}\right.$	*C*_3_
	$\left\{\begin{array}{l} {\hat{R}}_{jd}^{\left(5\right)},S_{A}=1\\ {\hat{R}}_{jd}^{\left(6\right)},S_{A}=0 \end{array}\right.$	*C*_4_–*C*_5_
*F*_2_	$\left\{\begin{array}{l} {\hat{R}}_{jd}^{\left(5\right)},i_{qc}>e_{2}\\ {\hat{R}}_{jd}^{\left(6\right)},i_{qc}<-e_{2} \end{array}\right.$	*C*_2_–*C*_3_
	$\left\{\begin{array}{l} {\hat{R}}_{jd}^{\left(5\right)},S_{A}=1\\ {\hat{R}}_{jd}^{\left(6\right)},S_{A}=0 \end{array}\right.$	*C*_4_–*C*_5_
*F*_3_	$\left\{\begin{array}{l} {\hat{R}}_{jd}^{\left(6\right)},i_{qc}>e_{2}\\ {\hat{R}}_{jd}^{\left(5\right)},i_{qc}<-e_{2} \end{array}\right.$	*C*_2_–*C*_3_
	$\left\{\begin{array}{l} {\hat{R}}_{jd}^{\left(5\right)},S_{B}=1\\ {\hat{R}}_{jd}^{\left(6\right)},S_{B}=0 \end{array}\right.$	*C*_4_–*C*_5_
*F*_4_	${\hat{R}}_{jd}^{\left(5\right)}$	*C*_1_–*C*_5_
*F*_5_	${\hat{R}}_{jd}^{\left(6\right)}$	*C*_1_–*C*_5_
*F*_6_	$\left\{\begin{array}{l} {\hat{R}}_{jd}^{\left(5\right)},S_{U}=1\\ {\hat{R}}_{jd}^{\left(6\right)},S_{U}=0 \end{array}\right.$	*C*_5_

**Table 4:** Health status classification description.

Grade	Corresponding Health Status	Insulation Resistance to Ground (Ω)
A	Fault	500 > *R_jd_* ≥ 0
B	Abnormal	2 k > *R_jd_* ≥ 500
C	Deterioration	20 k > *R_jd_* ≥ 2 k
D	Normal	*R_jd_* ≥ 20 k

## Data Availability

No new data were created or analyzed in this study. Data sharing is not applicable to this article.
